# Effect of *L. acidophilus* and *B.* *lactis* on blood glucose in women with gestational diabetes mellitus: a randomized placebo-controlled trial

**DOI:** 10.1186/s13098-019-0471-5

**Published:** 2019-08-28

**Authors:** Farnaz Sahhaf Ebrahimi, Aziz Homayouni Rad, Metanat Mosen, Fatemeh Abbasalizadeh, Aydin Tabrizi, Leila Khalili

**Affiliations:** 1 Obstetrics and Gynecology, Women’s Reproductive Health Research Center, Tabriz University of Medical Sciences, Tabriz, Iran; 2 Department of Food Science and Technology, Faculty of Nutrition and Food Sciences, Nutrition Research Center, Tabriz University of Medical Sciences, Tabriz, Iran; 3 Pediatric Neurology Research Center, Shahid Beheshti University of Medical Sciences, Tehran, Iran; 4 Department of Nutrition, Faculty of Nutrition and Food Sciences, Tabriz University of Medical Sciences, Tabriz, Iran

**Keywords:** Gestational diabetes mellitus, Probiotic, Yoghurt

## Abstract

**Background:**

Gestational diabetes mellitus (GDM) is a complication of pregnancy that can be associated with neonatal complications and adverse pregnancy outcomes. Recently, probiotic use has been proposed for better control of glucose in GDM patients. The aim of this study was to evaluate the effect of probiotic yoghurt compare with ordinary yoghurt on GDM women.

**Methods:**

In this double-blind placebo-controlled clinical trial, 84 pregnant women with GDM were randomly assigned into two groups of 42 recipients who underwent 300 g/day of probiotic yoghurt or placebo for 8 weeks. Blood glucose, HbA1c, and the outcome of pregnancy were compared between the two groups after the intervention.

**Results:**

According to the findings of present trial no significant differences were observed in general characteristics between the two groups (p > 0.05). Both fasting and post prandial blood glucose as well as the level of HbA1c were decreased significantly in probiotic group (p < 0.05), although these changes are not statistically significant in the placebo group. The between group differences was significant after the 2 month intervention (p < 0.05). Neonates born of probiotic group mothers, have significantly lower weight and fewer macrosome neonates were born in this group compared with control group (p < 0.05). However, no difference was observed in other values of outcome.

**Conclusions:**

Our study revealed that better control of blood glucose can be achieved by consumption of probiotic yoghurt in patients whose pregnancy is complicated by GDM, compared with placebo. Also incidence of macrosomia may be decreased by this regimen.

## Plain English summary

Present trial conducted to evaluate the effect of probiotic yoghurt containing *L. acidophilus* and *B.* *lactis* compare with ordinary yoghurt containing *S. thermophilus* and *L. bulgaricus* on GDM women. In this double-blind placebo-controlled clinical trial, 84 pregnant women with GDM were randomly assigned into two groups of 42 recipients who underwent 300 g/day of probiotic yoghurt or placebo for 8 weeks. Biochemical parameters and the outcome of pregnancy were compared between the two groups after the intervention. The results of this study revealed that better control of blood glucose can be achieved by consumption of probiotic yoghurt in patients whose pregnancy is complicated by GDM, compared with placebo. Also incidence of macrosomia may be decreased by this regimen.

## Background

Gestational diabetes mellitus (GDM) is known as a complication of pregnancy and is characterized by glucose intolerance which leads to adverse events such as macrosomia, neonatal hypoglycemia, neonatal hyper-bilirubinemia, preterm labor and increased risk of cesarean section [1, 2]. About 7% of all pregnancies in the United States are complicated by GDM, and its prevalence in Iran is approximately 6% of pregnancies [3, 4]. Recently, various complementary therapies have been considered for controlling blood glucose. Probiotics are microorganisms which can produce a microbial balance in the intestine and have a positive effect on the host [5–8]. Some of the mostly documented health benefits for probiotics include effectiveness against diarrhea, improvement of lactose metabolism, immunomodulation, as well as anti-inflammatory, anti-carcinogenic, anti-diabetic, hypo-cholesterolemic, and hypotensive characteristics [6, 9–11]. In addition to their impact on gastrointestinal disorders, the effect of probiotics on the improvement of blood glucose and lipid profile in patients with type 2 diabetes mellitus and GDM has been reported [12–15]. It has been also reported that probiotics reduce blood glucose and improve insulin resistance in diabetic rats and humans [11, 16]. Despite of the importance of GDM and its impacts on maternal and neonatal outcomes, few studies have evaluated the probiotics effect on improving glucose intolerance and insulin resistance as well as the outcomes of pregnancies complicated by GDM. Considering the potential of probiotic bacteria, the aim of the present trial was to investigate the effects of probiotic yoghurt containing *L. acidophilus* and *B.* *lactis* consumption on the glycemic parameters including FBG, post prandial BS, and HbA1c and the outcome of pregnancy including gestational age, weight, length, head circumference, macrosomia, and admission to NICU in GDM patients.

## Methods

### Subjects

In this double-blind placebo-controlled clinical trial, 84 patients with the diagnosis of GDM were recruited consecutively from the outpatient obstetrics clinic of Tabriz University of Medical Sciences. Inclusion criteria was as the following: patients referring to Tabriz Al-Zahra and Talegani high-risk outpatient clinic with the diagnosis of GDM, patients in their second trimester of pregnancy and patients diagnosed by oral glucose tolerance test (OGTT) between 24th and 28th weeks of pregnancy. Exclusion criteria was presence of other physical or psychological problems, presence of already-known fetal anomalous and not to consent to involve in the study.

### Sample size

The sample size for the study was calculated on the basis of the results (mean ± SD) for FBG as reported by Ejtahed et al. [12] with a confidence level of 95% and a power of 80%. Taking into account the probable dropout of patients during the intervention course as well as those who may not adhere to the study protocol, 42 patients with GDM were recruited for each group.

### Study design

Subjects were randomly assigned to the probiotic group (n = 20) receiving 300 mg/day of probiotic yoghurt (contained 10^6^ *Lactobacillus acidophilus* and *Bifidobacterium lactis*) or placebo (n = 20) group receiving 300 mg/day of ordinary yoghurt for 8 weeks, using a block randomization procedure with stratified subjects in each block based on age and week of pregnancy. All cans were coded by the company (Pegah Dairy Industries Company) and either the researcher or the patients were unaware of the contents. One week before the beginning of the trial, all patients refrained from eating yoghurt or any other fermented foods. All patients were asked, throughout the 8-week trial, to maintain their usual dietary habits and lifestyle and to avoid consuming any yoghurt other than that provided to them by the researchers and any other fermented foods. The patients were instructed to keep the yoghurt under refrigeration and to avoid any changes in medication, if possible.

This study was approved by the Ethics Committee of Tabriz University of Medical Sciences (Iran) and written informed consent was obtained from all subjects before inclusion in the study.

### Clinical and biochemical measurements

At baseline, all participants were examined by an obstetrician and the parameters including age, history of pregnancies, weight, height, body mass index, smoking, and blood pressure were measured. Ten ml of venous whole blood was obtained from each participant both before and after intervention after 12-h overnight fasting. The primary outcomes were the level of fasting blood glucose (FBG), post prandial blood glucose (BG), and HbA1c. Additionally, the secondary outcomes were the neonatal outcomes including weight, length, head circumference, presence of macrosomia and need for NICU admission that were also recorded.

### Statistical analysis

Statistical analysis was performed using SPSS software version 18.0 (SPSS, Inc., USA). Normality of variables distribution was evaluated using the Kolmogorov–Smirnov test. Variables not normally distributed were analyzed using nonparametric tests. Categorical and normally distributed quantitative variables were displayed as numbers (percentages) and mean ± SD, respectively. Non-normally distributed quantitative variables were presented as median (interquartile range). Between groups comparisons were made by χ^2^, independent-sample *t* test, and paired sample t test, as appropriate. Correlations between variables were analyzed by Pearson correlation test or Spearman rank correlation analysis. *p *< 0.05 was considered statistically significant.

## Results

### Characteristics of patients

**Fig. 1:**
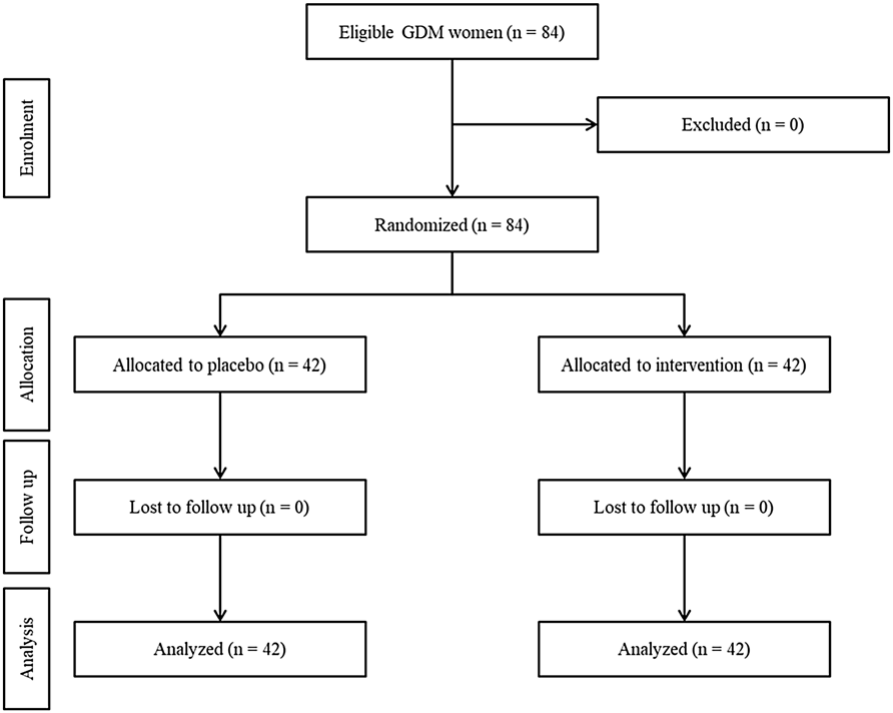
Summary of patient flow diagram

As revealed in the study flow diagram (Fig. 1), 84 pregnant women [probiotic (n = 42) and placebo (n = 42)] completed the trial. General characteristics of study subjects are showed in Table 1. The mean ± SD age of all participants was 31.6 ± 5.7 years. The mean ± SD weight, height and body mass index were 79.2 ± 11.5 kg, 161.8 ± 5.1 cm and 30.7 ± 4.5 kg/m^2^ respectively. The mean ± SD systolic and diastolic blood pressures were 111.4 ± 6.6 and 71.9 ± 5.5 mmHg respectively. As shown, No significant differences were observed in general characteristics between two groups.

**Table 1:** Characteristics of patients

Characteristics	Probiotic yoghurt group (n = 42)	Conventional yoghurt group (n = 42)	*p* value*
Age (year)	31.64 ± 5.97	31.61 ± 5.49	0.98
History of GDM (n (%))	0	4(9.5)	0.11
Smoking (n (%))	0	4(9.5)	0.11
Weight (kg) (mean ± SD)	79.5 ± 17.31	73.73 ± 17.74	0.13
Height (cm) (mean ± SD)	161.32 ± 4.98	161 ± 4.64	0.76
BMI (kg/m^2^) (mean ± SD)	31.67 ± 5.44	29.67 ± 3.03	0.06
SBP (mmHg) (mean ± SD)	111.66 ± 4.89	111.05 ± 63	0.66
DBP (mmHg) (mean ± SD)	71.19 ± 4.52	72.63 ± 6.44	0.24
Activity			
Light (n (%))	11 (26.2)	5 (11.9)	0.09
Heavy (n (%))	31 (73.8)	37 (88.1)	
FBG (mg/dl)	97.1 ± 9.4	96.4 ± 10.4	0.24
HbA1c (mmol/mol)	5.65 ± 0.67	5.86 ± 1.12	0.48

*GDM* gestational diabetes mellitus, *SD* standard deviation, *BMI* body mass index, *SBP* systolic blood pressure, *DBP* diastolic blood pressure, *FBG* fasting blood glucose, *HbA1c* glycated hemoglobin

* *p* values indicate comparison between groups (χ^2^ or independent-sample *t* test, as appropriate)

### Primary outcomes

Table 2 evaluates the findings related to the level of blood glucose. As shown, both fasting and post prandial blood glucose as well as the level of HbA1c is decreased significantly in probiotic group, although these changes are not statistically significant in the placebo group. Moreover the between group differences were statistically different after 2 weeks intervention.

**Table 2:** Level of blood glucose and glycemic response

Variable	Probiotic yoghurt group (n = 42)	Conventional yoghurt group (n = 42)	p value
FBG (mg/dl)			
Before	97.1 ± 9.4	96.4 ± 10.4	0.241
After	94 ± 8.5	97.6 ± 14.3	0.048
p value*	0.013	0.36	
Post prandial BS (mg/dl)			
Before	144.3 ± 26.8	136.8 ± 23.7	0.452
After	123.9 ± 16.2	136.8 ± 18.7	0.002
p value*	< 0.001	0.95	
HbA1c (mmol/mol)			
Before	5.65 ± 0.67	5.86 ± 1.12	0.488
After	5.48 ± 0.62	5.76 ± 1.02	0.025
p value*	< 0.001	0.092	

*FBG* fasting blood glucose, *BG* blood glucose, *HbA1c* glycated hemoglobin

For baseline between group comparisons, p-values are based on independent t-test

For after intervention between group comparisons, p-values and confidence intervals are based on analysis of covariance

* For within group comparisons, *p* values and confidence intervals are based on paired t-test

### Secondary outcomes

Table 3 evaluates differences in neonatal outcomes between the two groups. As shown, neonates born to probiotic group mothers have significantly lower weight and fewer macrosome neonates were born in this group. However, no difference was observed in other values of outcome.

**Table 3:** Characteristics of neonates

Characteristics	Probiotic yoghurt group (n = 42)	Conventional yoghurt group (n = 42)	*p* value*
Gestational age (weeks)*	37.7 ± 1.9	38.1 ± 1.3	0.25
Weight (g)*	3105.7 ± 533.8	3435 ± 473.5	0.004
Length (cm)*	49.8 ± 3.5	50.5 ± 2.9	0.61
Head circumference (cm)*	36 ± 2.3	36.2 ± 2	0.76
Macrosomia**	2 (4.8)	8 (19)	0.04
Admission to NICU**	2 (4.8)	3 (7.1)	0.64

NICU, Neonatal intensive care unit

* Data are expressed as mean (SD) and p value based on independent t-test

** Frequency (percent) is reported and p value based on Chi-squared test

## Discussion

Management of GDM without any side effects by natural food is a challenge for medical nutrition therapy of GDM. The present research is the first study evaluated the effect of consumption of probiotic yoghurt containing *Lactobacillus acidophilus* and *Bifidobacterium lactis* on glycemic response the outcome of pregnancy in GDM patients. According to the findings of present study, we found that using probiotic yoghurt causes a significant improvement in blood glucose levels and reduce risk of macrosomia.

Throughout pregnancy the gut microbiota undergoes significant changes. From the first (T1) to the third trimester (T3), the species richness of the gut microbiome decreases [17], although this has not been observed in all studies [18]. There is an increase in *Proteobacteria* and *Actinobacteria* phyla and a reduction in beneficial bacterial species *Roseburia intestinalis* and *Faecalibacterium prausnitzii* [17, 19]. These changes in gut microbial composition cause inflammation and correlate with increases in fat mass, blood glucose, insulin resistance and circulating pro-inflammatory cytokines in the expectant mother [20]. This “diabetic-like” state observed during the later stages of all healthy pregnancies is thought to maximize nutrient provision to the developing fetus [21]. However, increased insulin resistance combined with an inability to secrete the additional insulin required to maintain glucose homeostasis can result in the development of gestational diabetes mellitus (GDM) in the mother and macrosomia in the baby. The fasting hyperglycemia in women with GDM is associated with increased short-term and long-term complications in neonates [22]. Safe and inexpensive interventions for prevention and treatment of GDM are needed. Considering that certain microorganisms in the gastrointestinal tract can produce a positive effect on host metabolism, probiotic supplements can help maintain bacterial diversity and homeostasis in people with metabolic disorders [22, 23]. Experiments involving human intubation and sampling of probiotics from the cecum showed that probiotics, when given in fermented milk, survive to the extent of 23.5% ± 10.4% of the administered dose. With the use of known probiotic species and strains, it was determined that the delivery of *Lactobacillus* *and* *bifidobacteria* *to* the cecum was ≈ 30% and 10% of the administered dose, respectively [24]. In a research conducted by Homayouni et al. in 2012, the made it clear that foods are better carriers for probiotics than supplements [25]. Considering the survivability of *Lactobacillus* *and* *bifidobacteria* in human gastric trac and by knowing that fermented dietary products are better vehicle for probiotics we have evaluated the efficacy of yoghurt containing *L. acidophilus* and *B.* *lactis* in patients whose pregnancy is complicated by GDM.

Several studies showed benefits of probiotic use for improving blood glucose control in patients with GDM and T2DM (type 2 diabetes mellitus) [9, 26–30]; however, the efficacy of probiotics on pregnancy outcomes in GDM patients was not studied before. The findings of present research indicated that consumption of probiotic yoghurt containing *L. acidophilus* and *B.* *lactis* for 2 months could improve glycemic control in women with GDM.

Asemi et al. [29] evaluated the effects of daily consumption of probiotic yoghurt on insulin resistance and levels of insulin in the serum of pregnant women in the third trimester of gestation. The probiotic yoghurt used in this study was enriched with a probiotic culture of *L. acidophilus LA5* and *Bifidobacterium animalis BB12* with at least 10^7^ Colony Forming Unities. Daily consumption of probiotic yoghurt for 9 weeks was effective in maintaining normal serum insulin levels in pregnant women and thus contributing to prevent the development of insulin resistance, which usually develops during the last trimester in pregnant women. The study demonstrated an improvement in glycemic control during the last trimester of pregnancy, extending in the postpartum period for 12 months.

In the study conducted by Badehnoosh et al. [31] on 60 subjects with GDM they found that consumption of probiotic capsule containing *Lactobacillus acidophilus*, *Lactobacillus casei* and *Bifidobacterium bifidum* (2 × 10^9^ CFU/g each) for 6 weeks had beneficial effects on glycemic response, and serum inflammatory and oxidative stress biomarkers.

Dolatkhah et al. [27] conducted a study with women between 18 and 45 years of age with GDM between 24 and 28 weeks of pregnancy. The study was based on the daily consumption of probiotic capsules containing four bacterial strains (4 × 10^9^ CFU) in lyophilized culture, or placebo. The probiotic supplement appeared to improve glucose metabolism and weight gain among pregnant women with GDM.

Karamali et al. [28] analyzed the effects of probiotic supplementation on glycemic control and the lipid profiles over a period of 6 weeks. This study included 60 pregnant women with GDM, from 24 to 28 weeks of pregnancy. The probiotic group took a daily capsule containing 10^9^ CFU/g *L. acidophilus*, *L. casei*, and *Bifidobacterium bifidum*. After 6 weeks of treatment with probiotics, glycaemia, triglycerides, and VLDL cholesterol concentration decreased compared with the placebo group. In another 12-week study in pregnant women, probiotic supplementation containing the same strains, concluded that the probiotics had a positive effect on the metabolism of insulin, triglycerides, biomarkers of inflammation, and oxidative stress [32].

Recently, Jafarnejad et al. [33] analyzed the effects of a mixture of probiotics (VSL#3) on the glycemic state and inflammatory markers in 72 GDM patients through a double blind and randomized controlled clinical trial. The study groups consumed either a probiotic or placebo capsules twice a day for 8 weeks. The study concluded that for women with GDM, a probiotic supplementation can modulate some of the inflammatory markers and improve glycemic control.

In the study of Lindsay et al. [34], 149 pregnant women older than 18 years, before 34 weeks of pregnancy, were divided between probiotic and placebo groups and the aim of their study was to investigate the effects of probiotic capsule contained 100 mg *Lactobacillus salivarius* on metabolic parameters and pregnancy outcomes in pregnant women with GDM. No significant differences were observed between the groups concerning the post-intervention fasting blood glucose and birth weight. In addition, Lindsay et al. [35] investigated the effects of probiotic supplementation on fasting maternal glycaemia in obese pregnant women with a Body Mass Index (BMI) of > 30 kg/m^2^ between 24 and 28 weeks of pregnancy. A probiotic or placebo capsule was ingested daily, each probiotic capsule containing 100 mg of lyophilised *Lactobacillus salivarius*. The study showed no effect of probiotic intervention during 4 weeks on glycaemia. Their findings were different maybe because of the use of other probiotic strain and/or different intervention duration.

In general, probiotics can improve glycemic control and neonatal outcomes of patients with GDM [36]; however, the mechanisms whereby probiotics alter glucose homeostasis are not completely understood. One proposed method is by the production of short chain fatty acids (SCFAs), generated as a by-product of bacterial fermentation of dietary fibers. SCFAs act as an energy source for intestinal cells and have been found to regulate the production of hormones affecting energy intake and expenditure such as leptin and grehlin [37]. The binding of SCFAs to G protein-coupled receptors GPR41 and GPR43 increases the intestinal expression of Peptide YY and Glucagon-like peptide-1 (GLP-1) hormones which act to reduce appetite by slowing intestinal transit time and increasing insulin sensitivity [19]. Another hypothesized mechanism of SCFA action includes reducing gastrointestinal permeability by up-regulating transcription of tight junction proteins, enhancing production of Glucagon-like peptide-2 (GLP-2) which promotes crypt cell proliferation, and reducing inflammation in colonic epithelial cells by increasing PPAR-gamma activation [38]. Maintenance of the integrity of the gut barrier minimizes the concentration of lipopolysaccharide (LPS) in circulation. LPS is a structural component of gram negative bacterial cell walls, which induces an immune-cell response upon absorption into the human bloodstream, stimulating pro-inflammatory cytokine production and the onset of insulin resistance and hyperglycemia [39].

Considering the beneficial effects of probiotic supplementation in present research and the less amount of studies in this field, further research are needed to investigate the beneficial effects of several probiotic strains in different dose and duration on biochemical parameters and pregnancy outcomes in GDM patients.

## Conclusion

In conclusion, our study revealed better control of blood glucose is achieved by consumption of probiotic yoghurt containing *L. acidophilus* and *B.* *lactis* in patients, whose pregnancy is complicated by GDM, compare with placebo. The positive effects of probiotics on glycemic control could be translated into favorable effect on decreasing the incidence of macrosomia.

The limitation of this study was the small sample size. A plan for more subjects, longer duration in the long term, and evaluating the effect of other probiotic strains is currently underway.

## Data Availability

Data are all contained within the paper.

## Abbreviations

GDM: gestational diabetes mellitus
HbA1c: hemoglobin A1c
OGGT: oral glucose tolerance test
FBG: fasting blood glucose
T2DM: type 2 diabetes mellitus
CFU: colony-forming unit
BMI: body mass index
SCFA: short chain fatty acid
GLP: glucagon-like peptide
PPAR: peroxisome proliferator-activated receptors
LPS: lipopolysaccharides

## References

[1] Group HSCR (2008). Hyperglycemia and adverse pregnancy outcomes. N Engl J Med.

[2] Tsai P-JS, Roberson E, Dye T (2013). Gestational diabetes and macrosomia by race/ethnicity in Hawaii. BMC Res Notes..

[3] Harlev A, Wiznitzer A (2010). New insights on glucose pathophysiology in gestational diabetes and insulin resistance. Curr Diab Rep.

[4] Almasi S, Salehiniya H (2014). The prevalence of gestational diabetes mellitus in Iran (1993–2013): a systematic review. J Isfahan Med Sch..

[5] Homayouni A (2009). Letter to the editor. Food Chem.

[6] Bastani P, Akbarzadeh F, Homayouni A, Javadi M, Khalili L (2016). Health benefits of probiotic consumption. Microbes in food and health.

[7] Rad AH, Mehrabany EV, Alipoor B, Mehrabany LV, Javadi M (2012). Do probiotics act more efficiently in foods than in supplements?. Nutrition..

[8] Vaghef-Mehrabany E, Homayouni-Rad A, Alipour B, Sharif S-K, Vaghef-Mehrabany L, Alipour-Ajiry S (2016). Effects of probiotic supplementation on oxidative stress indices in women with rheumatoid arthritis: a randomized double-blind clinical trial. J Am Coll Nutr.

[9] Khalili L, Alipour B, Asghari Jafar-Abadi M, Faraji I, Hassanalilou T, Mesgari Abbasi M (2016). The effects of *Lactobacillus casei* on glycemic response, serum Sirtuin1 and Fetuin-A levels in patients with type 2 diabetes mellitus: a randomized controlled trial. Iran Biomed J.

[10] Maleki Davood, Homayouni Aziz, Khalili Leila, Golkhalkhali Babak (2016). Probiotics in Cancer Prevention, Updating the Evidence. Probiotics, Prebiotics, and Synbiotics.

[11] Khalili L, Alipour B, Jafarabadi MA, Hassanalilou T, Abbasi MM, Faraji I (2019). Probiotic assisted weight management as a main factor for glycemic control in patients with type 2 diabetes: a randomized controlled trial. Diabetol Metab Syndr.

[12] Ejtahed H, Mohtadi-Nia J, Homayouni-Rad A, Niafar M, Asghari-Jafarabadi M, Mofid V (2011). Effect of probiotic yogurt containing *Lactobacillus acidophilus* and *Bifidobacterium lactis* on lipid profile in individuals with type 2 diabetes mellitus. J Dairy Sci.

[13] Zhang Q, Wu Y, Fei X (2015). Effect of probiotics on glucose metabolism in patients with type 2 diabetes mellitus: a meta-analysis of randomized controlled trials. Medicina..

[14] Yan Q, Li X, Feng B (2015). The efficacy and safety of probiotics intervention in preventing conversion of impaired glucose tolerance to diabetes: study protocol for a randomized, double-blinded, placebo controlled trial of the Probiotics Prevention Diabetes Programme (PPDP). BMC Endocr Disord.

[15] Ejtahed HS, Mohtadi Nia J, Homayouni Rad A, Niafar M, Asghari Jafarabadi M, Mofid V (2011). The effects of probiotic and conventional yoghurt on diabetes markers and insulin resistance in type 2 diabetic patients: a randomized controlled clinical trial. Iran J Endocrinol Metab.

[16] Yadav H, Jain S, Sinha P (2007). Antidiabetic effect of probiotic dahi containing *Lactobacillus acidophilus* and *Lactobacillus casei* in high fructose fed rats. Nutrition..

[17] Koren O, Goodrich JK, Cullender TC, Spor A, Laitinen K, Bäckhed HK (2012). Host remodeling of the gut microbiome and metabolic changes during pregnancy. Cell.

[18] DiGiulio DB, Callahan BJ, McMurdie PJ, Costello EK, Lyell DJ, Robaczewska A (2015). Temporal and spatial variation of the human microbiota during pregnancy. Proc Natl Acad Sci.

[19] Tilg H, Moschen AR (2015). Food, immunity, and the microbiome. Gastroenterology.

[20] Gohir W, Whelan FJ, Surette MG, Moore C, Schertzer JD, Sloboda DM (2015). Pregnancy-related changes in the maternal gut microbiota are dependent upon the mother’s periconceptional diet. Gut Microbes..

[21] Wang Q, Würtz P, Auro K, Mäkinen V-P, Kangas AJ, Soininen P (2016). Metabolic profiling of pregnancy: cross-sectional and longitudinal evidence. BMC Med.

[22] Sekirov I, Russell SL, Antunes LCM, Finlay BB (2010). Gut microbiota in health and disease. Physiol Rev.

[23] Gregor MF, Hotamisligil GS (2011). Inflammatory mechanisms in obesity. Annu Rev Immunol.

[24] Bezkorovainy A (2001). Probiotics: determinants of survival and growth in the gut. Am J Clin Nutr.

[25] Rad AH, Mehrabany EV, Alipoor B, Mehrabany LV, Javadi M (2012). Do probiotics act more efficiently in foods than in supplements?. Nutrition..

[26] Ejtahed HS, Mohtadi-Nia J, Homayouni-Rad A, Niafar M, Asghari-Jafarabadi M, Mofid V (2012). Probiotic yogurt improves antioxidant status in type 2 diabetic patients. Nutrition..

[27] Dolatkhah N, Hajifaraji M, Abbasalizadeh F, Aghamohammadzadeh N, Mehrabi Y, Abbasi MM (2015). Is there a value for probiotic supplements in gestational diabetes mellitus? A randomized clinical trial. J Health Popul Nutr.

[28] Karamali M, Dadkhah F, Sadrkhanlou M, Jamilian M, Ahmadi S, Tajabadi-Ebrahimi M (2016). Effects of probiotic supplementation on glycaemic control and lipid profiles in gestational diabetes: a randomized, double-blind, placebo-controlled trial. Diabetes Metab.

[29] Asemi Z, Samimi M, Tabassi Z, Rad MN, Foroushani AR, Khorammian H (2013). Effect of daily consumption of probiotic yoghurt on insulin resistance in pregnant women: a randomized controlled trial. Eur J Clin Nutr.

[30] Li C, Li X, Han H, Cui H, Peng M, Wang G (2016). Effect of probiotics on metabolic profiles in type 2 diabetes mellitus: a meta-analysis of randomized, controlled trials. Medicine..

[31] Badehnoosh B, Karamali M, Zarrati M, Jamilian M, Bahmani F, Tajabadi-Ebrahimi M (2018). The effects of probiotic supplementation on biomarkers of inflammation, oxidative stress and pregnancy outcomes in gestational diabetes. J Matern Fetal Neonatal Med.

[32] Jamilian M, Vahedpoor Z, Dizaji SH (2016). Effects of probiotic supplementation on metabolic status in pregnant women: a randomized, double-blind, placebo-controlled trial. Arch Iran Med.

[33] Jafarnejad S, Saremi S, Jafarnejad F, Arab A (2016). Effects of a multispecies probiotic mixture on glycemic control and inflammatory status in women with gestational diabetes: a randomized controlled clinical trial. J Nutr Metab.

[34] Lindsay KL, Kennelly M, Culliton M, Smith T, Maguire OC, Shanahan F (2014). Probiotics in obese pregnancy do not reduce maternal fasting glucose: a double-blind, placebo-controlled, randomized trial (Probiotics in Pregnancy Study). Am J Clin Nutr.

[35] Lindsay KL, Brennan L, Kennelly MA, Maguire OC, Smith T, Curran S (2015). Impact of probiotics in women with gestational diabetes mellitus on metabolic health: a randomized controlled trial. Am J Obstet Gynecol.

[36] Rad AH, Abbasalizadeh S, Vazifekhah S, Abbasalizadeh F, Hassanalilou T, Bastani P (2017). The future of diabetes management by healthy probiotic microorganisms. Curr Diabetes Rev.

[37] Kellow NJ, Coughlan MT, Reid CM (2014). Metabolic benefits of dietary prebiotics in human subjects: a systematic review of randomised controlled trials. Br J Nutr.

[38] Jayashree B, Bibin Y, Prabhu D, Shanthirani C, Gokulakrishnan K, Lakshmi B (2014). Increased circulatory levels of lipopolysaccharide (LPS) and zonulin signify novel biomarkers of proinflammation in patients with type 2 diabetes. Mol Cell Biochem.

[39] Cani PD, Amar J, Iglesias MA, Poggi M, Knauf C, Bastelica D (2007). Metabolic endotoxemia initiates obesity and insulin resistance. Diabetes.

